# Development of a numerical model to predict physiological strain of firefighter in fire hazard

**DOI:** 10.1038/s41598-018-22072-8

**Published:** 2018-02-26

**Authors:** Yun Su, Jie Yang, Guowen Song, Rui Li, Chunhui Xiang, Jun Li

**Affiliations:** 1 College of Fashion and Design, Donghua University, Shanghai, 200051 China; 2 Iowa State University, Ames, 50010 Iowa USA; 3 Key Laboratory of Clothing Design and Technology, Donghua University, Ministry of Education, Shanghai, 200051 China

## Abstract

This paper aims to develop a numerical model to predict heat stress of firefighter under low-level thermal radiation. The model integrated a modified multi-layer clothing model with a human thermoregulation model. We took the coupled radiative and conductive heat transfer in the clothing, the size-dependent heat transfer in the air gaps, and the controlling active and controlled passive thermal regulation in human body into consideration. The predicted core temperature and mean skin temperature from the model showed a good agreement with the experimental results. Parametric study was conducted and the result demonstrated that the radiative intensity had a significant influence on the physiological heat strain. The existence of air gap showed positive effect on the physiological heat strain when air gap size is small. However, when the size of air gap exceeds 6 mm, a different trend was observed due to the occurrence of natural convection. Additionally, the time length for the existence of the physiological heat strain was greater than the existence of the skin burn under various heat exposures. The findings obtained in this study provide a better understanding of the physiological strain of firefighter and shed light on textile material engineering for achieving higher protective performance.

## Introduction

Skin burn is one of the most common thermal injuries in firefighting and emergency rescue. Heat stroke of life-threatening injury also usually occurs during the firefighting task. According to recent statistics of firefighter fatalities in the United States, 40 out of 68 deaths in 2015 resulted from heat stress or overexertion that led to heart attack or stroke^1^. Almost two-thirds of the firefighters over age 40 who died in 2015 was due to heart attacks or other cardiac events^1^. The level of physiological strain developed during the firefighting has an important effect on the physical capability, cognitive function, work tolerance, and even physical injuries and illness (e.g., heat stroke)^2,3^.

In the past, most studies in intensive radiation and flash fire focused on the skin burn injury. Thermal Protective Performance (TPP) tester was developed to evaluate thermal protective performance of fabric under a combination of radiant and convective heat exposure^4^. Radiative protective performance of fabric under high-level thermal radiation could be examined by using Radiant Protective Performance (RPP) tester^5^. The protective performance of fabric under low-level thermal radiation was evaluated based on a stored thermal energy tester regulated by ASTM F2731-11^6^. The results demonstrated that the skin burn injury depended not only on the type and intensity of heat source^7^, but also on the fabric’s category and configuration^8^, the air gap size^9,10^ and the fit and design features of clothing^11,12^.

However, prevention of the skin burn injury does not guarantee the definite security for firefighters, since the heat stroke can occur under no skin burn. The skin burn injury is generally induced by thermal radiation or heat source, while the human physiological strain associated with firefighting is caused by a combined effect of thermal environments (e.g. fires and radiation) and strenuous work^13^. As a result, firefighting protective clothing has different effects on the skin burn and the human physiological strain. The protective clothing can provide a positive effect on preventing the skin burn and the physiological strain by resisting heat transfer from thermal environment to human body. However, the protective clothing also increases the burden of wearers due to its heavy and low permeability characteristics, especially in hot environments^2,14^. Therefore, protective measures which aim at avoiding skin burn does not necessarily reduce the human physiological strain. Accordingly, there is an urgent need to develop a further understanding of the physiological responses of firefighter in thermal environment and take measures to reduce the heat strain.

The physiological strain of firefighter can be investigated based on human trials in a simulated operating state of firefighter. The physiological reaction, such as heart rate, oxygen consumption, blood lactate concentration, skin temperature and core temperature, were measured to assess the human physiological strain in thermal environment^15^. For example, Hemmatjo *et al*.^16^ assessed the effect of various hot environments (low heat, moderate heat and severe heat) on the firefighters’ physiological responses. The lab and field trials were performed by Annaheim *et al*.^17^ to examine the effect of environmental heat and physical activity on thermo-physiological responses. Fontana *et al*.^18^ investigated the effect of different fabric layers of protective clothing on thermo-physiological responses of human study participants. Considering the safety and well-being of the trial subjects, however, the human physiological strain was only examined in the absence of environmental heat or in relatively low intensity heat environments that are not enough to simulate the risk of heat stroke of firefighter in common fire scenes. Therefore, it is extremely important to develop a safe evaluation method for investigating the human physiological strain of firefighter in a simulated fire environment.

With the rapid development of computer technology, numerical simulation has been widely used to simulate heat transfer in clothing and predict body physiological strain in recent decades. Comparing to the laboratory simulation method, the numerical simulation method can remedy the experimental limitation, by studying the physiological reaction of firefighter under simulated fire environments, such as flash fire and thermal radiation. In previous applications, numerical models of heat transfer in firefighting protective clothing subjected to different fire hazards have been developed^19–22^. In the early 1970s, Morse *et al*.^19^ modeled the thermal response of protective clothing subjected to a JP-4 fuel fire that considered the effect of pyrolysis, ignition, and combustion of fabric. One of the most significant developments on heat transfer in firefighting protective clothing was the introduction of Torvi’s model^20^. This numerical model proved that one-dimensional model effectively simulated heat transfer in firefighting protective clothing. A clothing numerical model developed by Song *et al*.^21^ was employed to explain heat transfer in a configuration that realistically simulates the shape of human body. The model considered simulated fire nature, temperature dependent fabric properties and air gaps distribution^21^. In regard to the heat transfer in low-level thermal radiation, several efforts were made on model development. A multi-layer fabric model in low-level thermal radiation was developed to simulate the transmitted and stored thermal energy within protective clothing^22^.

However, these developed models only focused on heat transfer on human skin burn injury, and are generally coupled with skin bio-heat transfer and burn models for predicting skin burn injuries in fire hazards. The model of skin burn prediction simplified body thermal regulation and did not predict the human physiological reaction to thermal environment^23^. Considering the effect of heat stress on operation safety of firefighter^1^, the physiological strain can be a more critical index to determine the health and safety of firefighter than the skin burn. Thus, it is significant to develop a numerical model on heat transfer in firefighting protective clothing which is coupled with human thermal regulation for investigating the human physiological reaction in fire hazards. In addition, the fire hazards confronted by firefighter are usually divided into three categories: routine, hazardous and emergency^24^. Since statistics showed that most of firefighting assignments are performed under routine condition (low-level thermal radiation) for a relatively prolonged periods of time^25^, it is more reasonable to firstly consider the low-level thermal radiation.

Therefore, the objective of this study is to predict physiological thermal strain of firefighter in low-level thermal radiation while simultaneously monitoring the skin burn injury. A heat transfer model is developed that describes heat transfer from radiative thermal environment to human body through multilayer protective clothing. The model is coupled with an improved human thermal regulation effects. The developed model is used to evaluate the effect of thermal environment and clothing on the physiological thermal strain developed to firefighters during exposure.

## Mathematical model

**Figure 1:**
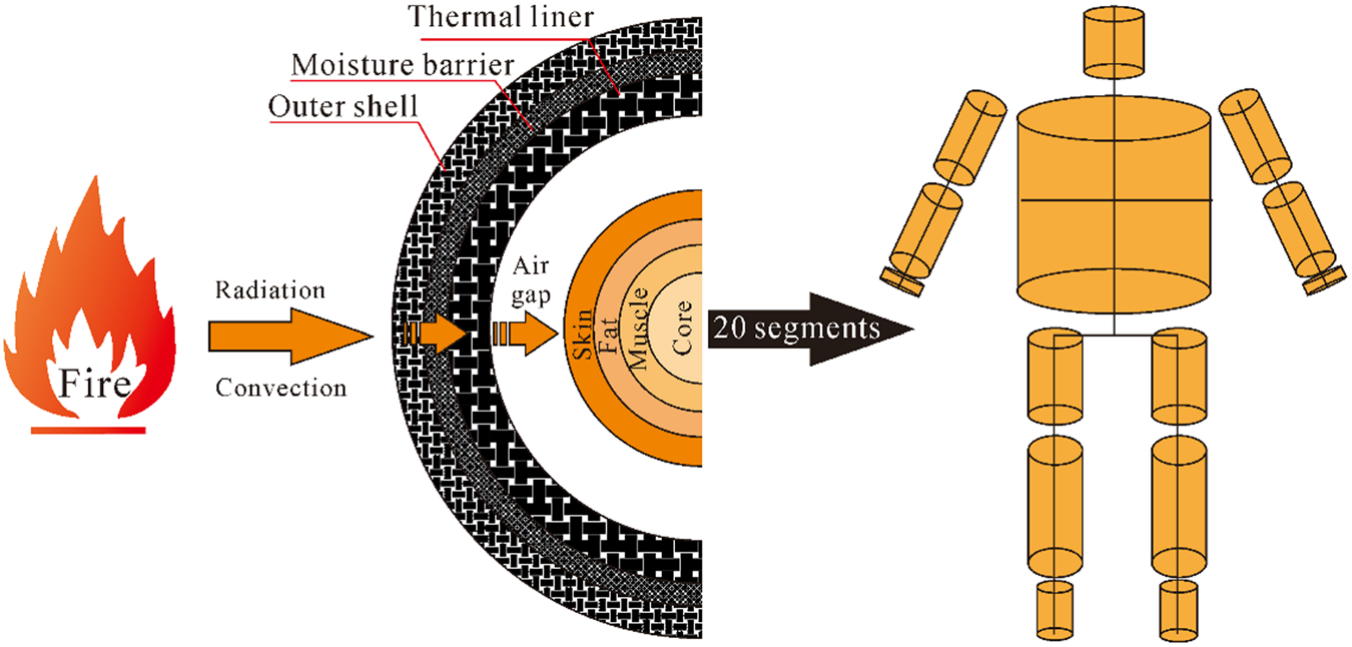
Schematic of heat transfer in “thermal environment– clothing– air gap– human body” system.

Firefighting protective clothing consists of outer shell, moisture barrier and thermal liner. In this work, a multilayer protective clothing system with air gaps of various sizes is focused. A radiant thermal environment is simulated with a nominal heat flux of 8.5 kW/m^2^, as is shown in Fig. 1^16^. A multi-node thermal regulation model is developed to simulate heat transfer in human body. The model incorporates the human body into four-layer structure including skin, fat, muscle and core. A human body is divided as 20 body parts according to body physiological characteristics and the 20-zone manikin ‘Newton’ (Thermetrics, Seattle, USA)^26^. Model assumptions are given as below to simplify the formulation:

1. Thermal energy from heat source to outer shell is transmitted in radiant mode. The radiative and convective heat loss between clothing and the surrounding environment is considered.
2. The clothing model is one-dimensional heat transfer model along the thickness direction of the clothing layers.
3. The radiative and conductive heat transfer is coupled in the clothing model; while the effect of convective heat transfer within the clothing layer is neglected^21^.
4. The thermal properties of the clothing layers are taken to be a function of temperature, but the optical properties of the clothing layers, such as absorptivity, reflectivity and transmissivity, are assumed as constant values.
5. The radiative and conductive heat transfer in the air gap between thermal liner and skin surface is considered. The occurrence of convective heat transfer depends on the air gaps size and temperature difference between the thermal liner and the skin surface.
6. Initial conditions are uniform throughout the clothing layer and the air gaps.
7. 20 segments of human body are exposed to the thermal radiation of same intensity.
8. The effects of evaporation heat loss from skin sweats, the increased blood flow rate due to high temperature condition, and the vasodilatation on the body heat transfer are simulated.

### Heat transfer in clothing layers

Heat exchange on the surface of the outer shell includes the radiant heat transfer from heat source, and the coupled convection and radiation between the outer shell and the surrounding environment. For cooling phase, there is no radiant heat transfer from the heat source. The heat exchange equations during the heat exposure and cooling phases are written as, respectively:

1 $$-{k}_{1}\frac{\partial T}{\partial x}{|}_{x=0}=-{h}_{conv,amb1}(T{|}_{x=0}-{T}_{amb})-\sigma {\varepsilon }_{1}{F}_{shell-amb}(1-{\varepsilon }_{g})({T}^{4}{|}_{x=0}-{{T}_{amb}}^{4})\quad 0 < t < {t}_{{\exp }}$$

2 $$-{k}_{1}\frac{\partial T}{\partial x}{|}_{x=0}=-{h}_{conv,amb2}(T{|}_{x=0}-{T}_{amb})-\sigma {\varepsilon }_{1}({T}^{4}{|}_{x=0}-{{T}_{amb}}^{4})\quad \quad \quad \quad \quad \quad \quad t > {t}_{{\exp }}$$

where *k*_1_ is the outer shell thermal conductivity; *h*_*conv*,*amb*1_ and *h*_*conv*,*amb2*_ are the convective heat transfer coefficient between the outer shell and the ambient during the exposure and the cool down phase, respectively; *T|*_*x=0*_ and *T*_*amb*_ are the temperature of the outer shell and the ambient, respectively; *ε*_1_ and *ε*_*g*_ are the outer shell emissivity (0.9) and the emissivity of hot gases (0.02)^20^, respectively; σ is the Stefan-Boltzmann constant, *F*_*shell-amb*_ is the view factor between the outer shell and the ambient, and *t*_*exp*_ is the exposure duration. The *h*_*conv*,*amb1*_ and *h*_*conv*,*amb2*_ in equations (1) and (2) are calculated by Nusselt number (*Nu*)^27^,

3 $${h}_{conv}=Nu\frac{{k}_{air}(T)}{L}$$

where *k*_*air*_ is the thermal conductivity of the air, *L* is the characteristic length of outer shell. According to the empirical correlation of free convection on a vertical plate^27^, the *Nu* is calculated as:

4 $$Nu=0.68+\frac{0.67R{a}^{1/4}}{{(1+{[0.492/\Pr ]}^{9/16})}^{4/9}}\quad \quad {10}^{-1} < Ra < {10}^{9}$$

5 $$Nu=0.825+\frac{0.387R{a}^{1/6}}{{(1+{[0.492/\Pr ]}^{9/16})}^{8/27}}\quad \quad \quad Ra > {10}^{9}$$

6 $$Ra=\frac{g\beta ({T}_{shell}-{T}_{amb}){L}^{3}}{\alpha v}$$

When the thermal energy is transmitted to the clothing layer, a portion of thermal energy is stored in the clothing system. The energy conservation equation of each clothing layer is given as below^20^:

7 $${(\rho {C}_{p})}_{fab}\frac{\partial T}{\partial t}=\frac{\partial }{\partial x}({k}_{fab}(T)\frac{\partial T}{\partial x})-\frac{\partial {q}_{rad-absorb}}{\partial x}$$

where *ρ*_*fab*_, (*C*_*p*_)_*fab*_ and *k*_*fab*_ are density of each fabric layer, specific heat and thermal conductivity, respectively; *q*_*rad-absorb*_ is the absorbed portion of the radiant heat flux from the heat source to the outer shell. Beer’s law is employed to account for the absorption of the incident thermal radiation (*q*_*rad-absorb*_) as it penetrates the pores of the fabric, i.e.^20^,

8 $${q}_{rad-absorb}={q}_{rad}\exp (-{\kappa }_{fab}x)$$

where Κ_*fab*_ is the extinction coefficient of the outer shell that is given by^27^

9 $${\kappa }_{fab}=In(\frac{1-r}{\tau })/{L}_{fab}$$

where τ is the transmissivity of outer shell and *L*_*fab*_ is the thickness of outer shell. The *q*_*rad*_ in equation (8) is the incident portion of the radiant heat flux from the heating source to the outer shell, which can be expressed as,

10 $${q}_{rad}=\frac{{F}_{hs-shell}\sigma ({\varepsilon }_{hs}{{T}_{hs}}^{4}-{\varepsilon }_{shell}{{T}_{shell}}^{4}){A}_{hs}}{{A}_{fab}}-\sigma {\varepsilon }_{shell}{F}_{shell-amb}(1-{\varepsilon }_{g})({{T}_{shell}}^{4}-{{T}_{amb}}^{4})$$

where *A*_*hs*_ and *A*_*fab*_ are the heating source area and the outer shell area, respectively; *T*_*hs*_ and *ε*_*hs*_ are the temperature of heating source and the emissivity of heating source, respectively; *F*_*hs-shell*_ is the fraction of the radiation leaving the heating source that strikes the outer shell.

The thermal conductivity of fabric is determined from the ratio of fiber to air fraction in the fabric as follows^20^.

11 $${k}_{fab}(T)=0.8{k}_{air}(T)+0.2{k}_{fiber}(T)$$

where *k*_*fiber*_ and *k*_*air*_ are the thermal conductivity of fiber and the thermal conductivity of air contained in the fabric’s pores, respectively, which are individually defined as follows^20^.

12 $${k}_{fiber}(T)={\rm{0.13}}+{\rm{0}}\mathrm{.0018}(T-300)$$

13 $${k}_{air}(T)={\rm{0.026}}+{\rm{0}}\mathrm{.000068}(T-\mathrm{300})$$

### Heat transfer in the air gap between clothing and human body

The heat transfer in the air gap plays an important role in thermal insulation of protective clothing, which is determined by the coupled heat transfer of convection, conduction and radiation. This model deals with the air gap as a radiation participating medium that only absorbs thermal radiation and does not emit thermal radiation. The heat transfer model within the air gap is written as follows^28^

14 $${(\rho {C}_{p})}_{air}\frac{\partial T}{\partial t}=\frac{\partial }{\partial x}({k}_{air}(T)\frac{\partial T}{\partial x})+\frac{\partial }{\partial x}{q}_{rad-absorb}$$

Where *ρ*_*air*_ and (*C*_*p*_)_*air*_ are the density and specific heat of air, respectively; and *q*_*rad-absorb*_ is the absorbed portion of the radiant heat flux from the thermal to the sensor. Beer’s law is employed to account for the absorption of the incident thermal radiation (*q*_*therm-sen*_), i.e.,

15 $${q}_{rad-absorb}={q}_{therm-sen}(1-\exp (-{\kappa }_{air}x))$$

where κ_*air*_ is the extinction coefficient of the air gap (5 m^−1^)^28^. The occurrence of convective heat transfer can be judged by calculating Rayleigh number (*Ra*), as shown in Equation (29). When the Ra is more than 1000, the natural convection in a vertical air gap is considered. The convective heat transfer in the air gap can be simulated in boundary conditions, which are written as,

16 $$-{k}_{therm}\frac{\partial T}{\partial x}|{}_{x={L}_{fab}}={q}_{therm-s{\rm{kin}}}+{h}_{conv-air}({T}_{therm}-{T}_{skin})$$

17 $$-{k}_{{ep}}\frac{\partial T}{\partial x}|{}_{x={L}_{{fab}}+{L}_{{air}}}={q}_{{therm}-{skin}}\exp ({\kappa }_{{air}}{L}_{{air}})+{h}_{{conv}-{air}}({T}_{{therm}}-{T}_{{skin}})$$

where *h*_*conv/cond*_ is the convective or conductive heat transfer coefficient in the air gap, which can be obtained by the equation (3). The air gap between thermal liner and skin surface can be treated as a rectangular enclosure. The *Nu* can be calculated by^29,30^:

18 $${Nu}=\{\begin{array}{ll}1.0, & \text{Ra}\,\le \,{{\rm{10}}}^{{\rm{3}}}\\ {\rm{0.22}}{(\frac{\Pr }{0.2+\Pr }\text{Ra})}^{{\rm{0.28}}}{(\frac{H}{{L}_{air}})}^{-{\rm{0.25}}}, & {{\rm{10}}}^{{\rm{3}}}\le \text{Ra}\le {{\rm{10}}}^{{\rm{10}}}\end{array}$$

### Human heat transfer and thermoregulation

Human body has a self-regulation function that can responds to varied thermal environments^31^. A multi-node thermal model is used to simulate human physiological responses under transient conditions with the input of human, clothing and environment parameters, such as activity intensity, clothing properties, ambient temperature, mean radiant temperature, relative humidity, and air velocity. The thermal regulation system consists of a controlling active system and a controlled passive system.

#### Passive system

The passive system simulates the heat transfer within body tissue layers, and between human body and the heat exposure environment through conduction, convection, radation, and evaporation^32^. The proposed thermoregulatory model^26^ divides the human body into 20 segments as that of the thermal manikin, including face, head, upper arms, forearms, hands, chest, shoulder, stomach, back, hips, thighs, calves, and feet. Each body segment has four layers: core, muscle, fat, and skin. In addition, the central blood compartment represented the blood circulation, making a total of 81-nodes^26^. Therefore, the proposed thermoregulatory model was also called 81-node model. The heat balance of each node (core, muscle, fat, and skin), except the central blood compartment, can be described by the following equations^33^:

19 $${c}_{i,1}\frac{d{T}_{i,1}}{dt}={Q}_{i,1}-{B}_{i,1}-{D}_{i,1}-{\rm{Re}}\,{s}_{i,1}$$

20 $${c}_{i,2}\frac{d{T}_{i,2}}{dt}={Q}_{i,2}-{B}_{i,2}+{D}_{i,1}-{D}_{i,2}$$

21 $${c}_{i,3}\frac{d{T}_{i,3}}{dt}={Q}_{i,3}-{B}_{i,3}+{D}_{i,2}-{D}_{i,3}$$

22 $${c}_{i,4}\frac{d{T}_{i,4}}{dt}={Q}_{i,4}-{B}_{i,4}+{D}_{i,3}-({R}_{i,4}+{C}_{i,4}+{E}_{i,4})$$

where *i* (from 1 to 20) refers to the body segments; *j* (1, 2, 3, 4) refers to the four layers of core, muscle, fat, and skin, respectively; The left terms in Eqs 19~22 are the rate of heat storage of core, muscle, fat, and skin, respectively; *c*_*i*,*j*_ is the heat capacity; *T*_*i*,*j*_ is the body temperature; *Q*_*i*,*j*_ is the rate of heat production; *B*_*i*,*j*_ is the heat exchange between each node and the central blood node; *D*_*i*,*j*_ is the heat exchange by conduction to the neighbor layer within the segment; Re*s*_*i*,*j*_ is the heat loss by respiration; and *C*_*i*,4_, *R*_*i*,4_ and *E*_*i*,4_ are the heat loss through convection, radiation, and evaporation from skin, respectively.

The heat balance of the central blood compartment is calculated as follows^26^:

23 $${c}_{81}\frac{d{T}_{81}}{dt}=\sum _{i=1}^{20}\sum _{j=1}^{4}{B}_{i,j}$$

where, the left term in Eq. 23 is the heat storage rate of the central blood compartment; the right term in Eq. 23 is the total heat exchange between each node and the central blood node through convection; *c*_81_ and *T*_81_ are the heat capacity and the temperature of the central blood compartment, respectively.

#### Active system

The active system simulates vasodilatation, vasoconstriction, sweating, and shivering using warm and cold signals, which are received by the warm and cold receptors, respectively^33^. Those signals are calculated by the temperature difference between each node and its set point (*Err* = *T* − *T*_*set*_). If *Err* > 0 (vasodilatation and sweating occur), then *Wrm* = *Err*, *Cld* = 0. If *Err* < 0 (vasoconstriction and shivering occur), then *Cld* = −*Err*, *Wrm* = 0. Here *Wrm* and *Cld* are the warm and cold signals, respectively. The weighted warm skin signals (*Wrms*) and cold skin signals (*Clds*) are used as the control variables to regulate physiological reaction. The control equations for vasodilatation, vasoconstriction, sweating and shivering are expressed as follows, respectively:

24 $${D}_{L}={C}_{dl}Err(1)+{S}_{dl}(Wrms-Clds)+{P}_{dl}Wrm(1)Wrms$$

25 $${S}_{T}=-{C}_{st}Err(1)-{S}_{st}(Wrms-Clds)+{P}_{st}Cld(1)Clds$$

26 $${S}_{w}={C}_{sw}Err(1)+{S}_{sw}(Wrms-Clds)+{P}_{sw}Wrm(1)Wrms$$

27 $${C}_{h}=-{C}_{ch}Err(1)-{S}_{ch}(Wrms-Clds)+{P}_{ch}Cld(1)Clds$$

where *D*_*L*_, *S*_*T*_, *S*_*w*_, and *C*_*h*_ are the signals for vasodilatation, vasoconstriction, sweating, and shivering, respectively. *C*_*dl*_, *S*_*dl*_, *P*_*dl*_, *C*_*st*_, *C*_*h*_, *S*_*st*_, *P*_*st*_, *C*_*sw*_, *S*_*sw*_, *P*_*sw*_, *C*_*ch*_, *S*_*ch*_, and *P*_*ch*_ are the control coefficients. Based on the work of Stowijk^33^ and Tanabe *et al*.^34^, these control coefficients are modified according to the surface area and weight of each body segment. *Err*(1), *Wrm*(1), and *Cld*(1) are the error signal, warm signal, and cold signal of core layer.

### Numerical computation

The developed model is composed of transient partial differential and ordinary differential equations. A finite difference scheme is applied to obtain the numerical solution. The Crank-Nicholson implicit scheme is applied to discretize the transient partial differential equations based on a (non-uniform) grid spanning a 1D space coordinate and time coordinate. A non-linear tri-diagonal system is obtained by discretizing the boundary conditions. Due to nonlinearity that comes from the radiation boundary condition, the coupling conduction-radiation, and the variation in the fabric thermo-physical and convective heat transfer coefficient with temperature, the Gauss-Seidel point-by-point iterative scheme is employed to solve these discrete equations. The central difference method is applied to calculate the ordinary differential equation. Specifically, the model parameters and temperatures from the previous time step are used as initial values for the iteration loop. The space-step and time-step are defined as 5 × 10^−6^ m and 0.1 s, respectively.

## Experimental test

### Materials

Flame-resistant fabrics widely used in the firefighting protective clothing were selected to validate the developed model. The basic properties of the selected fabrics were shown in Table 1. The thickness of test specimens was measured in accordance with standard ASTM D 1777–96. The mass per unit area was tested based on an electronic scale tester, conformed with standard ASTM D3776-96. The laser pulse method and differential scanning calorimeter (NETZSCH DSC 204 F1) tests were employed to measure thermal conductivity and specific heat of the fabrics.

**Table 1:** Properties of the fabrics used in the numerical simulation.

Layer	Component	Thickness (mm)	Density (kg/m^3^)	Specific heat (J/(kg K))	Thermal conductivity (W/(m K))
Outer shell	100% Nomex	0.6	342	1570	0.047
Moisture barrier	80% Nomex/20% Kevlar (PTFE)	0.9	122	1160	0.034
Thermal liner	100% M-aramid	2.2	123	1350	0.035

### Methods

**Figure 2:**
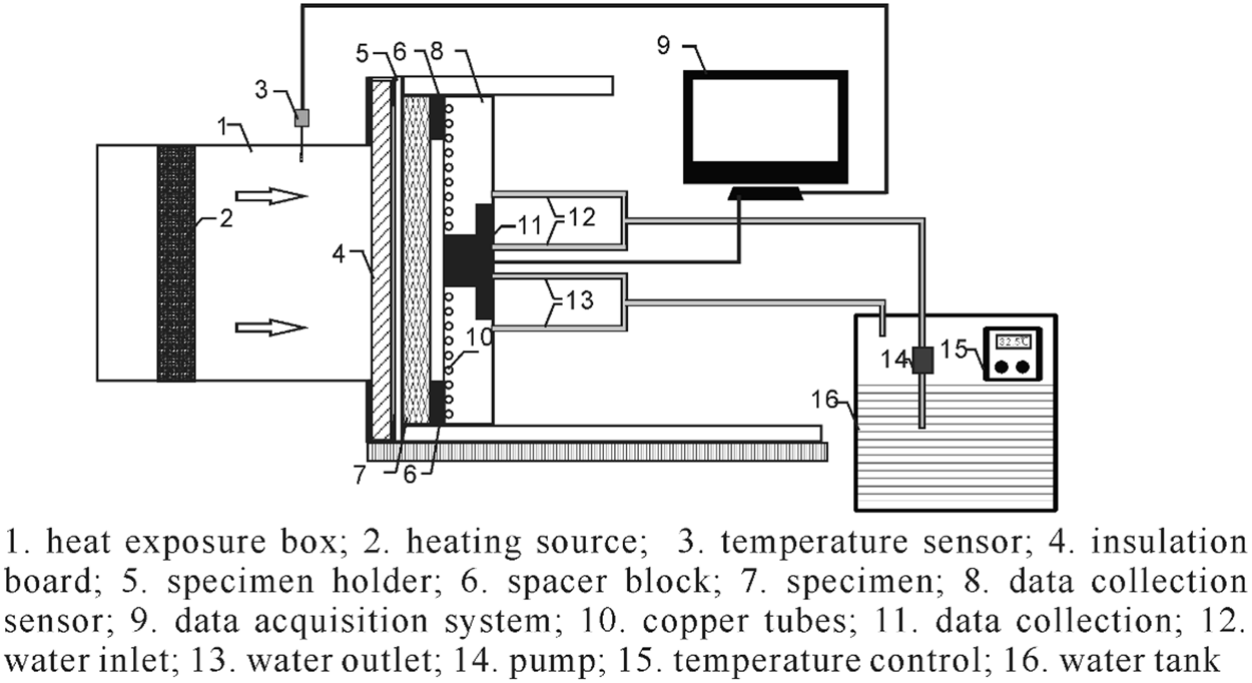
Diagram of heat transfer tester for low-level radiation^35^.

A bench top tester that measures skin heat flux in radiant heat exposure was used to validate the developed model, as shown in Fig. 2^35^. The radiant heat flux ranged from 0 to 21 kW/m^2^ can be produced by a black ceramic heat source. The skin heat flux was measured by a water cooled Schmidt-Boelter thermopile type sensor (Medtherm Corporation, USA). According to ASTM F2731-11, the parameters of heat source and skin-simulant sensor are given in Table 2. A 6.4 mm air gap between the clothing and the sensor assembly was simulated by inserting a 6.4 mm spacer.

**Table 2:** Basic parameters of the heating source and the sensor.

Parameter	Symbol	Unit	Value
Heating source temperature	T_hs_	K	733.15
Hot gases	ε_g_	—	0.02
Heating source area	A_hs_	m^2^	0.0144
Sensor area	A_sen_	m^2^	0.01
Heat source emissivity	ε_hs_	—	0.98
Sensor emissivity	ε_sen_	—	0.90

The fabric samples were conditioned in a constant temperature and humidity chamber (25 °C and 65% relative humidity) for at least 24 hours. The testing apparatus was firstly calibrated, and then the fabric system was modeled in a vertical orientation, subjected to a radiant heat source with a nominal heat flux of 8.5 kW/m^2^ for 300 s. The ambient temperature and relative humidity were approximately 30 °C and 65%, respectively. After the heat exposure, the skin-simulant sensor continued to record the skin heat flux for 300 s, as cooling phase. The obtained skin heat flux was treated as the boundary condition of human thermo-regulation model to calculate mean skin and core temperatures. Each specimen was tested three times and the average value was obtained.

## Results and Discussion

### Model verification

**Figure 3:**
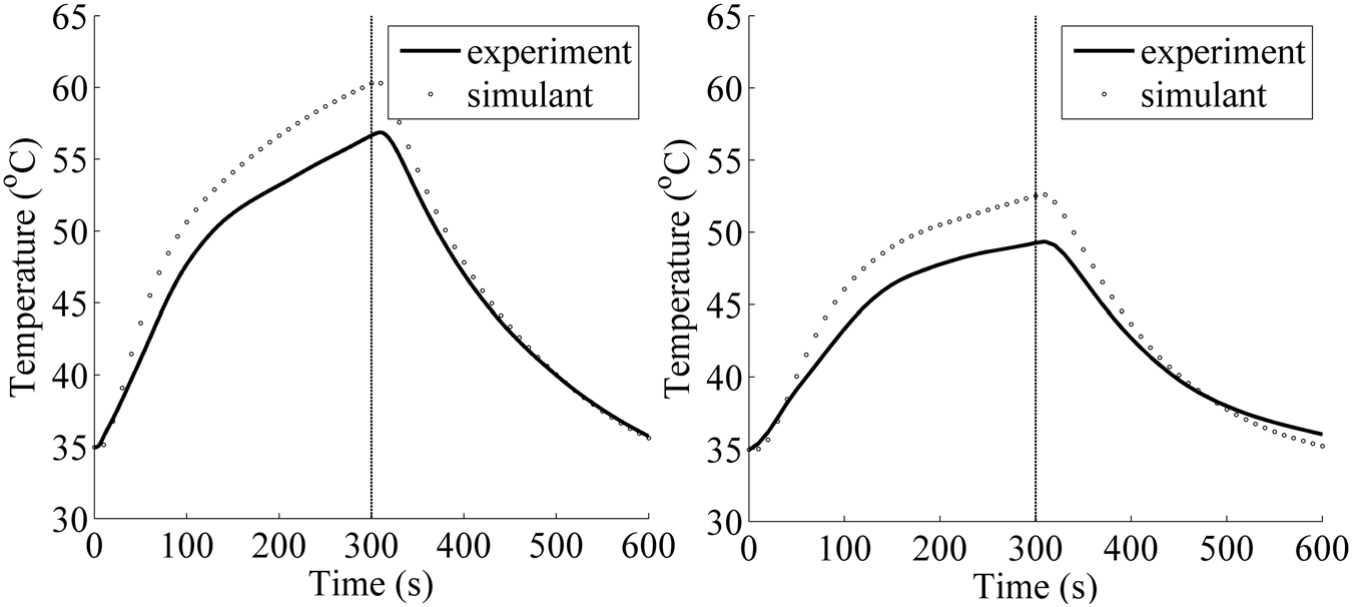
Comparison of mean skin temperature between the experiment and the simulant results: (**a**) without an air gap; (**b**) with an air gap.

Core temperature and mean skin temperature are two of the most important human physiological responses. The proposed model was applied to predict the core temperature and mean skin temperature of firefighter in a typical firefighting environment with a metabolic rate of 290 W/m^2 ^^36^. The predicted mean skin temperatures were compared with those from the experimental test, as shown in Fig. 3. The overall trend of simulant and experiment results presented a good agreement, indicating that the developed model could predict thermal response of human body in low level thermal radiation. In heat exposure of 300 seconds, the increasing rate of the mean skin temperature predicted by the model was larger than that from the experiment. The maximum predicted deviation for two cases (without and with air gap) were 3.63 and 3.26 °C, respectively. The difference could be attributed to ignorance of the multi-dimensional heat transfer and the coupled heat and moisture transfer in the clothing. It was reported that the central temperature for the heated region of protective fabric was higher than the surrounding temperatures, thus the thermal energy from the central zone of protective fabric could be transferred to the surrounding zone^37^. The moisture transfer in protective fabric presented a complex impact on the heat transfer in different heat exposure conditions^38^. However, a positive effect of moisture transfer was found in low intensity radiant exposure due to the heat storage and the evaporative heat loss of moisture^25^, which could decrease the temperature rise of skin. After the heat exposure of 300 seconds, the temperature difference between the model prediction and the experiment gradually decreased. This was due to the fact that the temperature difference between human skin and ambience determines the cooling rate of human body. Additionally, the moisture within the protective fabric increased the fabric’s heat capacity^25^. As a result, more stored thermal energy was discharged and reducing the cooling rate after the exposure.

**Figure 4:**
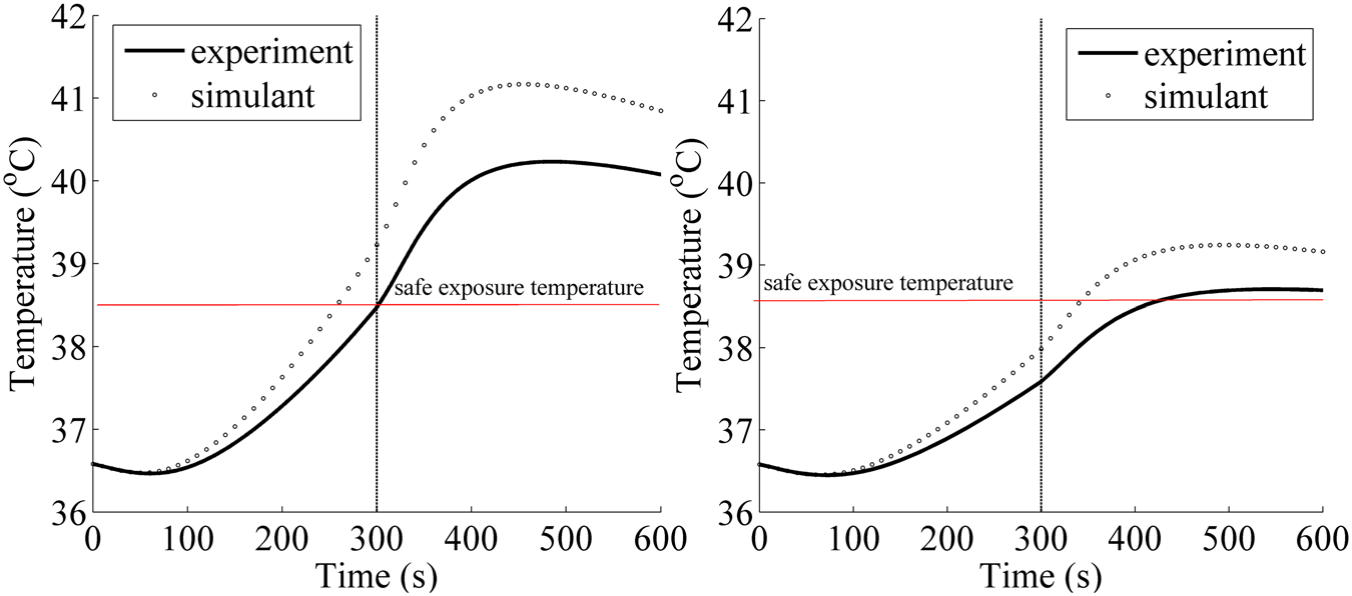
Comparison of the core temperature between the experiment and the simulated results: (**a**) without an air gap; (**b**) with an air gap.

Figure 4(a,b) show comparison of the core temperature prediction with the experiments in protective clothing without and with an air gap, respectively. A larger increase rate of the core temperature was observed for the model prediction. The core temperature continued to increase after the heat exposure of 300 seconds because of a lag effect of heat transfer from the clothing to human body. The differences of the core temperature between the model prediction and the experiment without and with an air gap were 0.75 and 0.40 °C, respectively. The core temperature normally maintains within 37.0 ± 1.0 °C^39^. The core temperature of less than 38.5 °C could be defined as safe exposure temperature^40,41^, and the corresponding time for the core temperature to stay at less than 38.5 °C was treated as safe exposure time. It was clear that the safe exposure time from the experiment without and with an air gap were 301.6 and 407.0 seconds, which presented a difference of 14.5% and 17.1% compared with the simulant results, respectively. For the clothing system without air gap, the difference of the safe exposure time was resulted from the core temperature difference during heat exposure. However, the core temperature difference during cooling phase increased the difference of the safe exposure time for the clothing system with an air gap.

### Effect of radiant intensity

**Figure 5:**
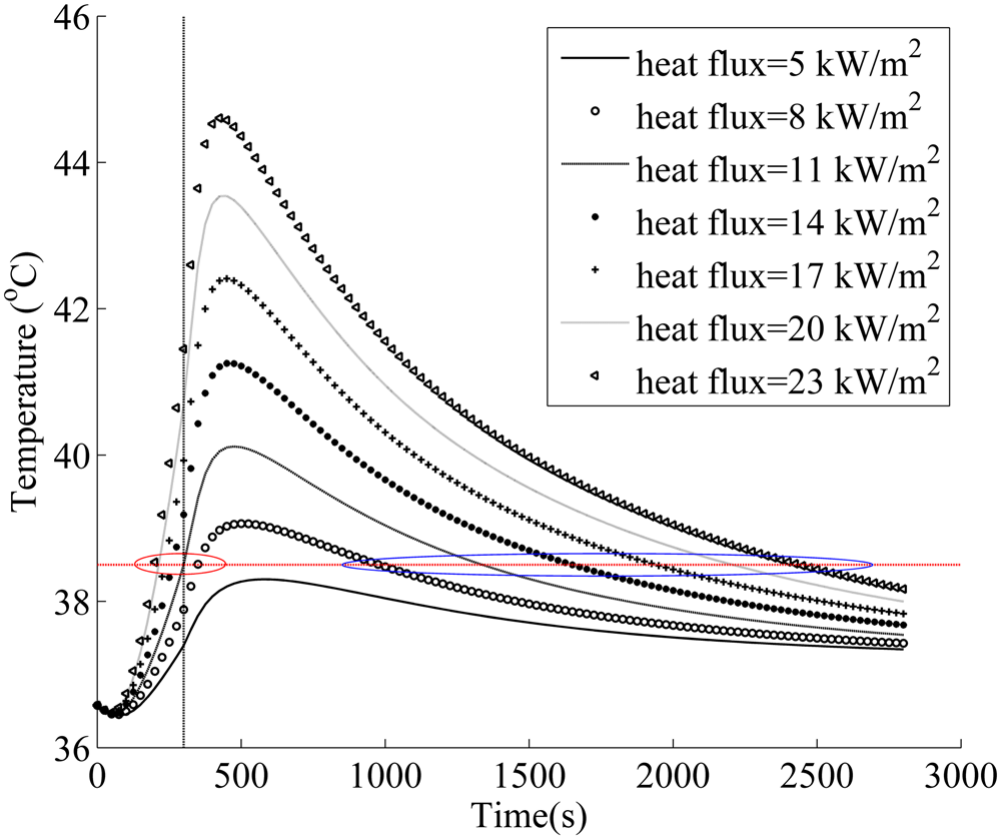
Change of core temperature over time in radiant heat exposure of 5 to 23 kW/m^2^. (Note: the red line indicates the limit value of human physiological strain^40,41^; the intersections in red wireframe indicate the safe exposure time; the intersections in blue wireframe indicate the effective cooling time).

Firefighters in fire-fighting and emergency rescue are usually exposed to low-level thermal radiation that ranges from 2.1 to 21 kW/m ^26^. In order to obtain the safe exposure time of firefighter in different intensity of heat exposure, the proposed model was used to predict core temperature in radiant heat exposure of 5 to 23 kW/m^2^, as shown in Fig. 5. The exposure and cooling times in the simulation were 300 and 2500 seconds, respectively. It was found that the rise of radiant exposure intensity increased the core temperature of human body. The highest core temperature did not occur in the heat exposure phase, but in the cooling phase. This was because the discharge of stored thermal energy in the clothing layer and the delay effect of heat transfer continued to increase the core temperature^22^. After around 500 seconds, the core temperature began to decrease due to the heat loss to the cooling environment. In the cooling phase, the core temperature showed an increase with the rising of radiant heat flux, while the difference of core temperature between different heat fluxes reduced over time.

**Figure 6:**
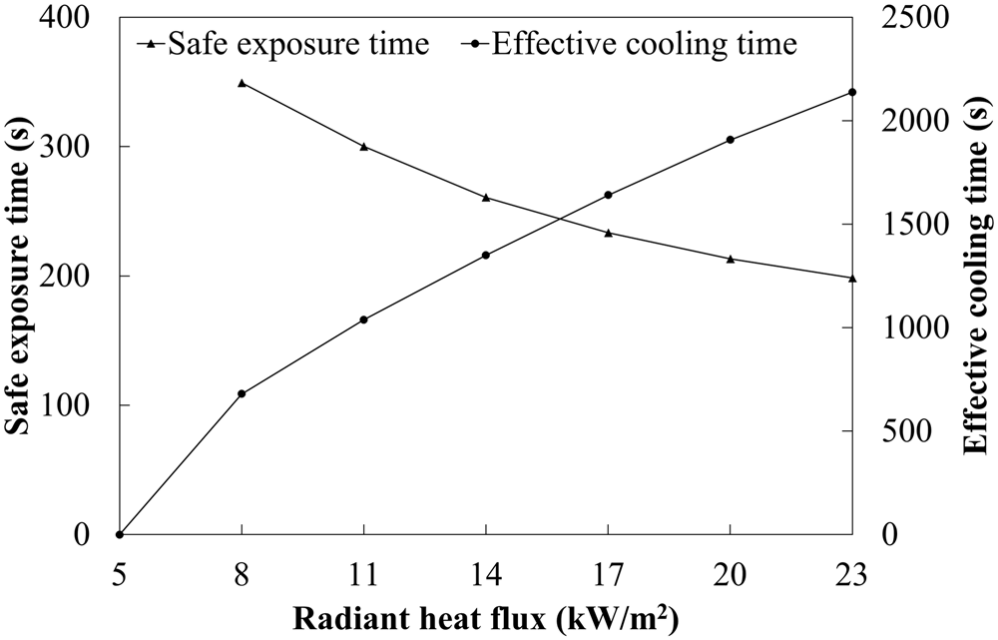
Change of safe exposure time and effective cooling time with radiant heat flux.

According to the above discussion, firefighter could be subjected to heat stress when the core temperature is above 38.5 °C. The time range in which the core temperature reaches above 38.5 °C is defined as dangerous time zone of firefighter^40,41^. The dangerous time zone in different heat exposures can be found in Fig. 5. The dangerous time zones start from the safe exposure time and end by the effective cooling time. Figure 6 illustrates the variation of safe exposure time and effective cooling time against radiant heat flux. The core temperature for the radiant heat flux of 5 kW/m^2^ was found to be less than 38.5 °C during the heat exposure and cooling phases. This indicated that the protective clothing could provide effective thermal protection for firefighter in radiant heat flux of 5 kW/m^2^. The increased radiant heat flux reduced the safe exposure time and increased the effective cooling time. But the change rates of safe exposure time was relatively less than that of effective cooling time. This indicated that the radiant heat flux had greater influence on the effective cooling time. The result illustrated that the cooling effect in rest environment of 30 °C could not instantly take firefighters out of danger. For radiant heat flux of 23 kW/m^2^, firefighter would need at least 2136 seconds to recover to normal state after the heat exposure of 300 seconds.

**Figure 7:**
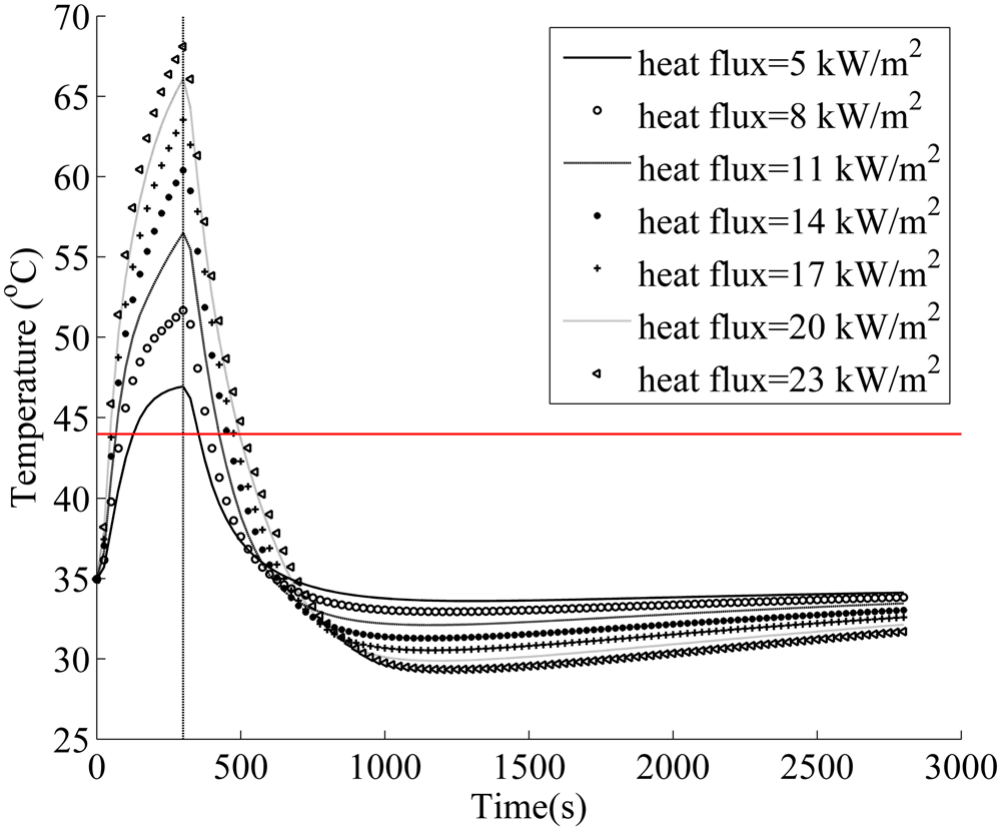
Change of mean skin temperature with time in radiant heat exposure of 5 to 23 kW/m^2^. (Note: the red line indicates the threshold value of skin burn)^43^.

Figure 7 shows the effect of radiant heat flux on the mean skin temperature of firefighter wearing a clothing system with an air gap. The difference of the mean skin temperature between different radiative heat fluxes gradually reduced with the increase of radiant heat flux. After heat exposure, the mean skin temperature reduced rapidly especially for the higher heat flux. Thus, the change of the mean skin temperature presented an obviously different behavior from the change of core temperature. The mean skin temperature was less than 44 °C after 500 seconds, indicating that the skin layer did not continue to produce burn since 44 °C is a threshold value of skin burn^42^. These results revealed that the physiological heat strain of human body lasted for longer time than skin burn in radiant heat exposure.

### Effect of air gap size

**Figure 8:**
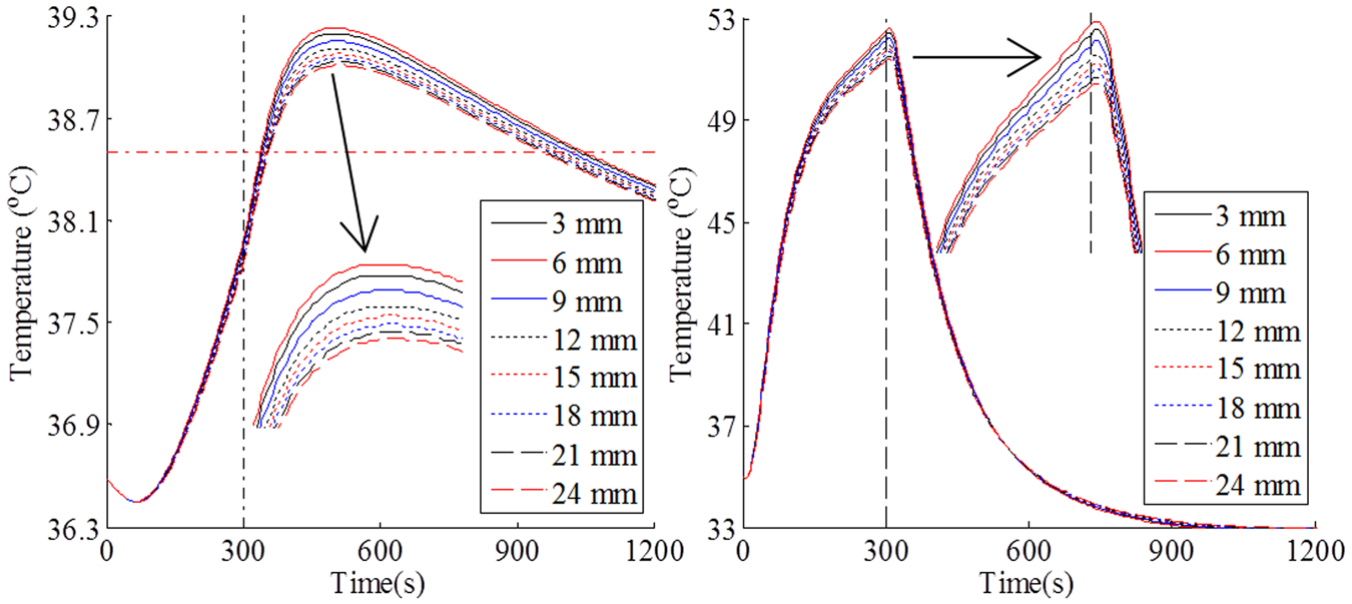
Changes of core temperature (**a**) and mean skin temperature (**b**) with time in an air gap of 0 to 24 mm. (Note: the red dot dash line indicates the limit value of human physiological strain)^40,41^.

**Figure 9:**
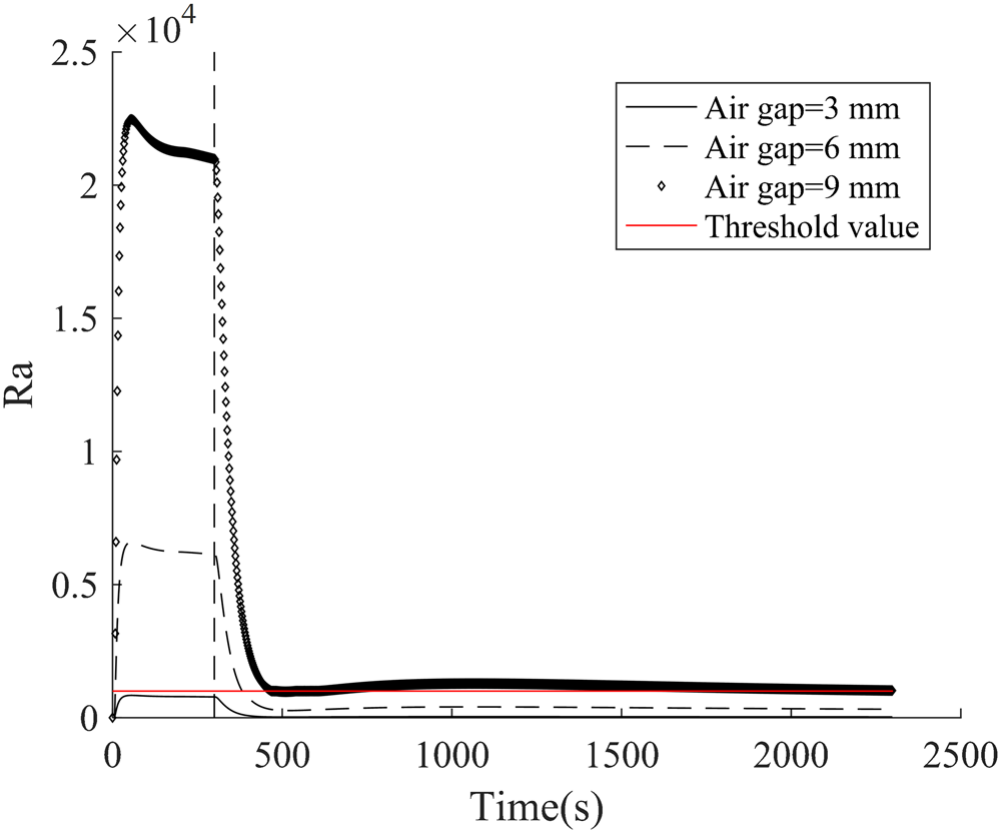
Change of Ra with time in an air gap of 3 to 9 mm. (Note: the red line indicates the threshold value for the occurrence of convection heat transfer^45^).

Due to the much lower thermal conductivity, air gap between the clothing and the human body can provide high thermal insulation against fire hazards. However, the thermal insulation effect could be overshadowed by the natural heat convection effect with an increase of the size of the air gap. It was reported that the average air gap size between the clothing and the body is around 25 mm^9^. Therefore, the air gap ranging from 0 to 24 mm was used in the developed model for investigating the relationship between the air gap size and the physiological strain of human body. Figure 8(a,b) show the effect of air gap size on core temperature and mean skin temperature predicted by numerical model, respectively. Figure 8 does not presented the tempeature changes for 0 mm air gap since the core and mean skin temperatures for 0 mm air gap were obviously higher than that for other air gaps. This indicated that the existence of an air gap of small size had a significant effect on preventing heat strain. Therefore,

When the air gap size was increased from 3 to 24 mm, the core temperature and mean skin temperature increased firstly and then decreased. For clothing with a 6 mm air gap, the peak values for both core temperature and mean skin temperature were the largest among all air gaps. The increased air gap size, while reducing the thermal conductivity, could also increase the convective heat transfer rate from the inner side of clothing to the human body. The natural convection in air gaps depends not only on the size and orientation of the air gap, but also on the intensity of heat exposure^43^. Previous studies reported the occurrence of natural convection in a horizontal air gap with a size of 6–7 mm^44^. The corresponding Rayleigh number (Ra) in the air gap was more than 1708^45^. For the vertical air gap, however, the convective heat transfer occurred when Ra was more than 1000^45^. Figure 9 shows the change of Ra in a vertical air gap with time. It was clear that Ra increased greatly with the increase of the size of air gap. The convective heat transfer during heat exposure initiated in an air gap that is more than 3 mm. It could continue to exist in the air gaps of larger than 9 mm in the cooling phase. In addition, the air gap size had an important effect on the cooling effect of human body. The air gap resisted heat loss from the human body to the surrounding environment. When there is no air gap, the core temperature and mean skin temperature presented the rapidest decrease during the cooling. The temperature difference between the different air gap sizes gradually decreased with cooling time.

**Figure 10:**
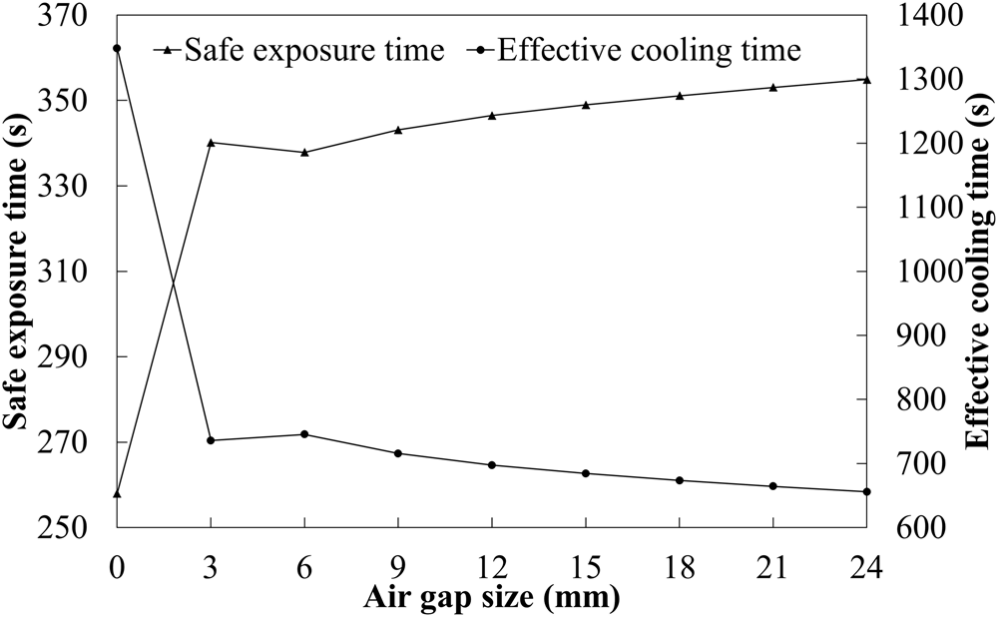
Change of safe exposure time and effective cooling time with air gap size.

As can be seen from Fig. 8(a), the core temperatures of different air gap sizes were higher than the limit value of human physiological strain. According to the intersection of core temperature and safe exposure temperature, the safe exposure time and the effective cooling time over air gap size are obtained and showed in Fig. 10. As the air gap size increased, the safe exposure time showed an increase, while the effective cooling time obviously decreased. The safe exposure time of air gap of 24 mm was 1.38 times that of no air gap. The effective cooling time of no air gap was more than twice that of air gap of 24 mm. This indicated that the increase of air gap size could reduce the dangerous time zone of core temperature. However, when the air gap size was larger than 6 mm, the safe exposure time and effective cooling time presented an opposite tendency against the air gap size. As discussed above (see Fig. 9), the convective heat transfer occurred in an air gap of 6 mm, which could enhance heat transfer between protective clothing and human body. Although the convective heat transfer rate gradually increased with the air gap size, the radiative and conductive heat transfer showed a decrease^45^. Therefore, the safe exposure time continued to increase with the increase of the air gap size when the size exceeded 6 mm.

## Conclusions

A numerical model that consists of both heat transfer in firefighting protective clothing and human thermal regulation was developed to predict physiological heat strain of firefighter in low-level thermal radiation. The model was validated from the experiment simulation. Based on the parameter study of the model, it was found that the intensity of radiative heat exposure was an important factor influencing the human physiological strain. The increase of its intensity linearly shortened the safe exposure time and increased the effective cooling time. When the air gap size was smaller than 6 mm, the human physiological strain was reduced by the increased air gap size. However, the occurrence of convective heat transfer increased the total heat transfer from the clothing to the human body when the air gap size was equal or exceeding 6 mm. Thus, the developed model is capable of simulating heat transfer in multi-layered protective clothing with an air gap. It can be used to effectively predict the thermal response of firefighter in fire hazards. The findings in this study provide a fundamental understanding of material engineering and safe operation, and proper training of firefighter in fire hazards.
